# Supplementary material for: Associations of body fat percentage with C-reactive protein levels in Canadian adults with and without osteoarthritis: Findings from the Canadian Longitudinal Study on Aging (CLSA)
Source: PLoS One. 2026 Feb 26;21(2):e0341604. doi: 10.1371/journal.pone.0341604 (PMC12944775; doi:10.1371/journal.pone.0341604)
Supplement: S1 File — (DOCX) [file pone.0341604.s001.docx]

SOP_DCS_0021

Timed (4-metre) Walk Test. Canadian Longitudinal Study on Aging. <https://www.clsa-elcv.ca/doc/530>. Accessed November 11 2022.

SOP_DCS_0024

Chair Rise Test. Canadian Longitudinal Study on Aging. <https://www.clsa-elcv.ca/doc/533>. Accessed November 11 2022.

SOP_DCS_0023

Measuring Standing Balance. Canadian Longitudinal Study on Aging. <https://www.clsa-elcv.ca/doc/532>. Accessed November 11 2022.

SOP_DCS_0022

Times Get Up and Go Test. Canadian Longitudinal Study on Aging. <https://www.clsa-elcv.ca/doc/531>. Accessed November 11 2022.

SOP_BCP_0001

Collection of blood by venipuncture. Canadian Longitudinal Study on Aging. <https://www.clsa-elcv.ca/doc/501>. Accessed November 11 2022.

SOP_BCP_0003

Biospecimen post collection processing. Canadian Longitudinal Study on Aging. <https://www.clsa-elcv.ca/doc/503>. Accessed November 11 2022.

SOP_DCS_0028

Hand grip strength. Canadian Longitudinal Study on Aging. <https://www.clsa-elcv.ca/doc/537>. Accessed November 11 2022.

SOP_DCS_0017

Bone mineral density by dual- energy X-ray absorption (DXA) – whole body scan. Canadian Longitudinal Study on Aging. [https://www.clsa-elcv.ca/doc/526. Accessed November 11 2022](https://www.clsa-elcv.ca/doc/526.%20Accessed%20November%2011%202022).

Data Collection Site Questionnaires (Comprehensive). Canadian Longitudinal Study on Aging. <https://clsa-elcv.ca/doc/1122>. Accessed November 11 2022.

In-Home Questionnaire (Baseline – Comprehensive). Canadian Longitduinal Study on Aging. <https://clsa-elcv.ca/doc/1048>. Access November 11 2022.

Maintaining Contact Questionnaire (Tracking and Comprehensive) Wave 1 Version. <https://clsa-elcv.ca/doc/540>. Accessed November 11 2022.
