# Supplementary material for: Associations of body fat percentage with C-reactive protein levels in Canadian adults with and without osteoarthritis: Findings from the Canadian Longitudinal Study on Aging (CLSA)
Source: PLoS One. 2026 Feb 26;21(2):e0341604. doi: 10.1371/journal.pone.0341604 (PMC12944775; doi:10.1371/journal.pone.0341604)
Supplement: S2 File — (DOCX) [file pone.0341604.s002.docx]

| **Supplementary Table 1**. Demographic characteristics and baseline values for participants included in the statistical analyses for each of the 8 dependent variables. | | | | | | | | |
| --- | --- | --- | --- | --- | --- | --- | --- | --- |
| Dependent Variable | Females | | | | Males | | | |
|  | N | Outcome Mean (SD) | Age (years)  Mean (SD) | BMI (kg/m^2^)  Mean (SD) | N | Outcome Mean (SD) | Age (years)  Mean (SD) | BMI (kg/m^2^)  Mean (SD) |
| Grip Strength (kg) | 9007 | 25.7 (5.8) | 61.1 (9.7) | 27.6 (5.9) | 10059 | 42.2 (9.5) | 62.3 (10.0) | 28.1 (4.6) |
| Mobility Index | 9765 | 0.4 (2.7) | 61.3 (9.8) | 27.7 (5.9) | 10491 | 0.4 (2.6) | 62.4 (10.0) | 28.2 (4.6) |
| DXA Whole Body Fat Mass (g) | 9477 | 29161.9 (10308.9) | 61.2 (9.7) | 27.7 (5.9) | 10161 | 24916.4 (8639.0) | 62.4 (10.0) | 28.2 (4.6) |
| DXA Whole Body Fat Percent (%) | 9477 | 38.8 (6.2) | 61.2 (9.7) | 27.7 (5.9) | 10161 | 27.9 (5.4) | 62.4 (10.0) | 28.2 (4.6) |
| DXA Whole Body Lean Mass (g) | 9477 | 44246.6 (6963.0) | 61.2 (9.7) | 27.7 (5.9) | 10161 | 62425.8 (8704.6) | 62.4 (10.0) | 28.2 (4.6) |
| DXA Trunk Fat Percent (%) | 9477 | 36.6 (7.5) | 61.2 (9.7) | 27.7 (5.9) | 10161 | 29.0 (6.4) | 62.4 (10.0) | 28.2 (4.6) |
| DXA Appendicular Fat Mass Index (kg/m^2^) | 9477 | 5.4 (1.8) | 61.2 (9.7) | 27.7 (5.9) | 10161 | 3.3 (1.1) | 62.4 (10.0) | 28.2 (4.6) |
| DXA Appendicular Lean Mass Index (kg/m^2^) | 9477 | 6.9 (1.1) | 61.2 (9.7) | 27.7 (5.9) | 10161 | 8.8 (1.1) | 62.4 (10.0) | 28.2 (4.6) |
| Note. *BMI*, body mass index; *DXA*, Dual-Energy X-Ray Absorptiometry. | | | | | | | | |
