# Supplementary material for: Associations of body fat percentage with C-reactive protein levels in Canadian adults with and without osteoarthritis: Findings from the Canadian Longitudinal Study on Aging (CLSA)
Source: PLoS One. 2026 Feb 26;21(2):e0341604. doi: 10.1371/journal.pone.0341604 (PMC12944775; doi:10.1371/journal.pone.0341604)
Supplement: S3 File — (DOCX) [file pone.0341604.s003.docx]

| **Supplementary Table 2**. Number (n) of participants for each dependent variable (grip strength, mobility index, DXA whole body fat mass, DXA whole body fat percent, DXA whole body lean mass, DXA trunk fat percent, DXA appendicular fat mass index, DXA appendicular lean mass index), separated by sex (females, males) and subgroup (A through H). | | | | | | | | |
| --- | --- | --- | --- | --- | --- | --- | --- | --- |
| FEMALES | Subgroup N | | | | | | | |
|  | A | B | C | D | E | F | G | H |
| Grip Strength (kg) | 3796 | 2623 | 577 | 687 | 314 | 353 | 238 | 419 |
| Mobility Index | 3977 | 2806 | 605 | 756 | 371 | 426 | 282 | 542 |
| DXA Whole Body Fat Mass (g) | 3868 | 2730 | 576 | 732 | 362 | 417 | 268 | 524 |
| DXA Whole Body Fat Percent (%) | 3868 | 2730 | 576 | 732 | 362 | 417 | 268 | 524 |
| DXA Whole Body Lean Mass (g) | 3868 | 2730 | 576 | 732 | 362 | 417 | 268 | 524 |
| DXA Trunk Fat Percent (%) | 3868 | 2730 | 576 | 732 | 362 | 417 | 268 | 524 |
| DXA Appendicular Fat Mass Index (kg/m^2^) | 3868 | 2730 | 576 | 732 | 362 | 417 | 268 | 524 |
| DXA Appendicular Lean Mass Index (kg/m^2^) | 3868 | 2730 | 576 | 732 | 362 | 417 | 268 | 524 |
| MALES | Subgroup N | | | | | | | |
|  | A | B | C | D | E | F | G | H |
| Grip Strength (kg) | 4766 | 3347 | 549 | 690 | 171 | 228 | 97 | 211 |
| Mobility Index | 4908 | 3479 | 563 | 734 | 187 | 267 | 107 | 246 |
| DXA Whole Body Fat Mass (g) | 4776 | 3357 | 547 | 711 | 178 | 253 | 104 | 235 |
| DXA Whole Body Fat Percent (%) | 4776 | 3357 | 547 | 711 | 178 | 253 | 104 | 235 |
| DXA Whole Body Lean Mass (g) | 4776 | 3357 | 547 | 711 | 178 | 253 | 104 | 235 |
| DXA Trunk Fat Percent (%) | 4776 | 3357 | 547 | 711 | 178 | 253 | 104 | 235 |
| DXA Appendicular Fat Mass Index (kg/m^2^) | 4776 | 3357 | 547 | 711 | 178 | 253 | 104 | 235 |
| DXA Appendicular Lean Mass Index (kg/m^2^) | 4776 | 3357 | 547 | 711 | 178 | 253 | 104 | 235 |
| Note. *DXA*, Dual-Energy X-Ray Absorptiometry. | | | | | | | | |
