# Supplementary material for: Associations of body fat percentage with C-reactive protein levels in Canadian adults with and without osteoarthritis: Findings from the Canadian Longitudinal Study on Aging (CLSA)
Source: PLoS One. 2026 Feb 26;21(2):e0341604. doi: 10.1371/journal.pone.0341604 (PMC12944775; doi:10.1371/journal.pone.0341604)
Supplement: Attachment — Submitted filename: Response to Reviewers_PLOS One.docx [file pone.0341604.s004.docx]

All changes to the manuscript are **bolded** in the Revised Manuscript document.

Comments to the Author

1. Is the manuscript technically sound, and do the data support the conclusions?

The manuscript must describe a technically sound piece of scientific research with data that supports the conclusions. Experiments must have been conducted rigorously, with appropriate controls, replication, and sample sizes. The conclusions must be drawn appropriately based on the data presented.

Reviewer #1: Yes

Reviewer #2: Yes

2. Has the statistical analysis been performed appropriately and rigorously?

Reviewer #1: I Don't Know

Reviewer #2: Yes

3. Have the authors made all data underlying the findings in their manuscript fully available?

The [PLOS Data policy](http://c05y1x9s.r.us-east-2.awstrack.me/L0/http:%2F%2Fwww.plosone.org%2Fstatic%2Fpolicies.action%23sharing/1/010f0191ba7e6107-b447f59b-4bf3-42e7-9854-d024c3cd22ce-000000/VsJTTXJXzedrduTvnNCV_YrwWrM=175) requires authors to make all data underlying the findings described in their manuscript fully available without restriction, with rare exception (please refer to the Data Availability Statement in the manuscript PDF file). The data should be provided as part of the manuscript or its supporting information, or deposited to a public repository. For example, in addition to summary statistics, the data points behind means, medians and variance measures should be available. If there are restrictions on publicly sharing data—e.g. participant privacy or use of data from a third party—those must be specified.

Reviewer #1: No

Reviewer #2: Yes

4. Is the manuscript presented in an intelligible fashion and written in standard English?

PLOS ONE does not copyedit accepted manuscripts, so the language in submitted articles must be clear, correct, and unambiguous. Any typographical or grammatical errors should be corrected at revision, so please note any specific errors here.

Reviewer #1: Yes

Reviewer #2: Yes

5. Review Comments to the Author

Please use the space provided to explain your answers to the questions above. You may also include additional comments for the author, including concerns about dual publication, research ethics, or publication ethics. (Please upload your review as an attachment if it exceeds 20,000 characters)

Reviewer #1: Summary

This study aims to explore the association between C-reactive protein (CRP) and physical function and body composition in CLSA participants with/without osteoarthritis (OA) and multimorbidity (MM). The analyses are disaggregated by sex, and linear regression is used where three models are compared: model 1 (covariates only, including subgroup defined by OA and MM categories), model 2 (covariates + log-CRP), and model 3 (covariates + log-CRP + log CRP x subgroup interaction). A change of 1%+ in R2 across the models is used to identify significance. Percentage adiposity measured by whole body fat percentage and trunk fat percentage was associated with elevated CRP (small effect size) in both females and males. Lower appendicular lean body mass was associated with elevated CRP (small effect size) in males only. These results were observed regardless of OA status, suggesting that percent adiposity and not OA is associated with systemic inflammation (measured by CRP).

Overall, this study is well done and the findings are quite interesting. To strengthen this paper, some of the methodological choices require further clarification/justification, and should as well be acknowledged as limitations, as noted in the detailed comments below.

Author Response/Action: Thank you for these comments and your well-considered suggestions to improve this manuscript.

Detailed Comments:

1. Abstract:
i. Objectives: Since all participants in the CLSA comprehensive database were included (complete cases) and the age range starts at 45 years, it may be misleading to refer to the participants in this study as ‘older’. It would be better to either state the age range explicitly (45-85 years), or remove ‘older’ as a qualifier.

Author Response: We agree and have made the following revisions outlined below.

Author Action: The ‘older’ qualifier was removed. The sentence now reads as follows: *“Determine the cross-sectional associations of serum inflammation with body composition and physical capacity in* ***~~older~~*** *Canadian****~~s~~*** ***adults*** *with/without osteoarthritis (OA) and multimorbidities.”* (line 33).

ii. Methods: It should be stated that analyses were sex disaggregated, as some results (appendicular lean mass index) held for only one sex (males).

Author Response: We agree and have made the following revisions outlined below.

Author Action: We added a statement to indicate that separate analyses for each sex were completed. The sentence now reads as follows: *“30,097 participants from the Canadian Longitudinal Study on Aging were categorized into eight subgroups (presence/absence of lower extremity OA, hand OA, multimorbidities)****, disaggregated by sex****.”* (lines 36-37).

Also, the criterion for deciding statistical significance (change in adjusted R-squared of 1%+ when CRP added to model with only covariates) should be included in the methods, as the results and interpretations are highly dependent on this choice (as noted in the manuscript, many more statistically-significant results were achieved [driven by the large sample)], but did not reach this threshold).

Author Response: We agree and have made the following revisions outlined below.

Author Action: We added a statement to define the threshold for model significance. The sentence now reads as follows: *“Linear regression models (multiple covariates and log-transformed CRP) were completed with body composition and physical function measures as dependent variables.* ***A priori, the research team defined that an increase in the adjusted R^2^ between models by 1% or more was considered significant.****”* (lines 39-40).

iii. Conclusions: It is stated that percentage adiposity and appendicular lean mass index were associated with CRP regardless of OA status. Given that the subgroups were defined by OA and MM status, what happened to MM? MM seems to be presented sporadically, which is confusing – e.g., MM is referred to in the title, referred to in the Methods and the Results, but not in the Conclusions. The manuscript reads this way as well – e.g., MM is not discussed in the Introduction, yet it appears later in the manuscript (Participant & Discussion, see comments below). The role of MM in this study is unclear and inconsistently presented.

Author Response: Thank you for highlighting this inconsistency. Although for this particular paper, we were more interested in the findings across different OA status, we agree that the findings across different multimorbidity status are equally as important and should therefore, be given equal attention. Therefore, we have made the following revisions outlined below.

Author Action:

*“Percent adiposity and appendicular lean mass index were associated with CRP regardless of OA* ***and multimorbidity*** *status, suggesting that percent adiposity, but not OA* ***or an accumulation of other chronic health conditions****, is associated with systemic inflammation.”* (lines 46-47).

*“Together, the systemic inflammation can be detected as elevated levels of the acute phase protein C-reactive protein (CRP) [12, 15-17]* ***which is elevated in many chronic health conditions [18-25]****. The level of circulating CRP is proportional to the intensity of the inflammatory process [****26****]”* (lines 75-76).

*“****~~A~~ While a*** *meta-analysis shows that CRP is higher in* ***~~people with~~*** *OA compared to healthy counterparts [****18****]****, there are other potential explanations for elevated systemic inflammation in those with OA. First, it is unclear if elevated CRP is linked with adiposity, a feature of body composition, commonly associated with OA; or if it is associated with OA disease and the joint tissue damage itself. Second, it is unclear if the relationship between CRP levels with physical function depends on the joint (knee, hip, hand) affected by OA. It is possible that OA in large weight-bearing joints, such as the hip and knee, contribute more to systemic inflammation compared to small joints in the hand. Third, ~~There is a stronger association between obesity and CRP in females compared to males [16,20].~~*** ***~~Furthermore,~~*** *in OA, elevated CRP concentrations are linked with (i) worse pain [****18-20****], (ii) greater disease incidence [****20****] and (iii) faster disease progression based on radiography [****21****], although results are somewhat inconsistent between studies [****18-21,27****].* ***~~Many questions about the importance of systemic inflammation in OA disease remain.~~*** ***However, the associations between CRP with measures of physical function, mobility and physical activity in OA are unknown. ~~First, it is unclear if elevated CRP is linked with adiposity commonly associated with OA, or if it is associated with OA disease and the joint tissue damage itself. Second, the associations between CRP with measures of physical function, mobility and physical activity in OA are unknown. Third, it is unclear if the relationship between CRP levels with physical function depends on the joint (knee, hip, hand) affected by OA.~~*** ***Fourth, those with OA are more likely to have one or more comorbidities (i.e. multimorbidity) compared to those without OA [28]; however it is unclear whether elevated CRP is associated with OA disease or with multimorbidity.****”* (lines 78-99).

2. Introduction:
i. Osteoarthritis (OA) and its linkages with obesity, systemic inflammation (measured by CRP), and physical function/mobility/physical activity appear to be the primary focus, as the current evidence and outstanding questions focus on these topics. There is no discussion of multimorbidity (MM) and it is unclear what role it plays in this study; the evidence linking MM with pain and physical function (in those with lower extremity OA) is only briefly mentioned in the Participants section of the paper and in the Discussion (where the evidence of the links between chronic conditions and inflammation are discussed). All of this evidence of MM should be moved to the Introduction, including the evidence linking chronic conditions (and by extension MM) to systemic inflammation.

Author Response: We agree and have made the following revisions outlined below.

Author Action:

*“Percent adiposity and appendicular lean mass index were associated with CRP regardless of OA* ***and multimorbidity*** *status, suggesting that percent adiposity, but not OA* ***or an accumulation of other chronic health conditions****, is associated with systemic inflammation.”* (lines 46-47).

*“Together, the systemic inflammation can be detected as elevated levels of the acute phase protein C-reactive protein (CRP) [12,15-17]* ***which is elevated in many chronic health conditions [18-25]****. The level of circulating CRP is proportional to the intensity of the inflammatory process [****26****].”* (lines 75-76).

*“****~~A~~ While a*** *meta-analysis shows that CRP is higher in* ***~~people with~~*** *OA compared to healthy counterparts [****18****]****, there are other potential explanations for elevated systemic inflammation in those with OA. First, it is unclear if elevated CRP is linked with adiposity, a feature of body composition, commonly associated with OA; or if it is associated with OA disease and the joint tissue damage itself. Second, it is unclear if the relationship between CRP levels with physical function depends on the joint (knee, hip, hand) affected by OA. It is possible that OA in large weight-bearing joints, such as the hip and knee, contribute more to systemic inflammation compared to small joints in the hand. Third, ~~There is a stronger association between obesity and CRP in females compared to males [16,20].~~*** ***~~Furthermore,~~*** *in OA, elevated CRP concentrations are linked with (i) worse pain [****18-20****], (ii) greater disease incidence [****20****] and (iii) faster disease progression based on radiography [****21****], although results are somewhat inconsistent between studies [****18-21,27****].* ***~~Many questions about the importance of systemic inflammation in OA disease remain.~~ However, the associations between CRP with measures of physical function, mobility and physical activity in OA are unknown. ~~First, it is unclear if elevated CRP is linked with adiposity commonly associated with OA, or if it is associated with OA disease and the joint tissue damage itself. Second, the associations between CRP with measures of physical function, mobility and physical activity in OA are unknown. Third, it is unclear if the relationship between CRP levels with physical function depends on the joint (knee, hip, hand) affected by OA.~~ Fourth, those with OA are more likely to have one or more comorbidities (i.e. multimorbidity) compared to those without OA [28]; however it is unclear whether elevated CRP is associated with OA disease or with multimorbidity.****”* (lines 78-99).

3. Methods:
i. Participants:
i. The analyses were done for complete cases. Please indicate the portion of the 30,000 comprehensive cohort participants that this represents (or the % of missing data pertaining to this cohort).

Author Response: This information has been added. See below for details.

Author Action: We have added the percentage of complete data. Depending on the specific dependent variable, this percentage ranged between 63% and 67%. The sentence now reads as follows: *“However, for each of the separate linear regression models, only data from participants with completed measurements of interest were included in the analyses* ***(63-67% depending on the variable of interest)****; those with partial data were excluded.”* (lines 141-142).

ii. Subgroups: A categorical variable is included among the covariates that has eight categories for the different combinations of OA and MM. MM is reflected as a binary variable (Yes if 2+ chronic conditions, No if < 2 chronic conditions), yet the stated evidence linking MM with pain and physical function pertains to the number of MM. Another way to model MM is to have this as a separate covariate with the measure being the number of chronic conditions (e.g., aligned with the stated evidence, increased sensitivity to participant differences in MM), and to have OA as its own (categorical) variable. Would this approach be a better way to capture MM (and OA) in the model?

Author Response: Thank you for this consideration regarding MM. There are multiple approaches to evaluate the association of MM with a specific outcome; for example, simple counts of the total number of comorbidities as Reviewer 1 proposed, weighted counts based on known associations of some conditions with the specific outcome, and weighted indices such as the Charlson Index [Nicholson, Almirall and Fortin, Health Psychology, 2019]. It is important to recognize that there is no consensus on a list of chronic health conditions that are associated with systemic inflammation. Thus, we selected the categorization of the presence versus absence of MM (the information available from the CLSA dataset) based on a common cutoff value of 2 conditions from the list of conditions captured in the CLSA dataset, as recommended by Nicholson and colleagues. This choice enabled us to account for the presence of MM while avoiding the challenges associated with some health conditions exerting a greater influence than others on CRP as a specific outcome (for example, Parkinson’s Disease likely shares a weaker relationship with CRP than heart disease).

Author Action: We have added statements to address the limitations associated with the inclusion of multimorbidity in the current study. The following statements have been added to the Limitations and Future Directions:

*“****Limitations associated with the statistical modelling included the (i) definition of multimorbidity as a binary variable (presence or absence); (ii) inclusion of subgroup as a covariate rather than separate analyses per group; and…****”* (lines 480-483).

*“****More sophisticated analyses that reflect the relative weight of different chronic health conditions and their association with CRP might yield different results, however there is no consensus on a list of chronic health conditions that are particularly associated with systemic inflammation.****”* (lines 484-487).

*“****Finally, an analysis involving a greater number of subgroups that further partition chronic conditions may provide a better opportunity to identify the nuances associated with markers of inflammation and their role in obesity-related multimorbidities.****”* (lines 508-511).

We have also revised the Methods section and added a reference: “*Participants were categorized as having multimorbidity if they self-reported a diagnosis of 2 or more of the listed 21 conditions* ***[30]****. A categorization of* ***the presence or absence*** ***~~2 or more multimorbidities~~*** *was selected based on previous evidence demonstrating an association between* ***~~the number of~~*** *multimorbidit****y~~ies~~****…*” (lines 159-161).

iii. Data and Statistical Analysis:
1. A change in the adjusted R2 of 1%+ is used to identify a significant result (due to the large sample sizes and resulting small p-values). While using the change in the adjusted R2 is a common approach to this problem, the choice of the threshold (1%+) requires justification; the results/interpretations are highly impacted by this choice, thus the rationale or supporting reference(s) for this threshold should be provided.

Author Response: We agree that the rationale for the chosen threshold should be presented more clearly. A 1% R^2^ value was selected as the minimum because an *r* value of 0.1 (or R^2^ of 0.01) represents a small effect (Cohen et al. 1992, doi: 10.1037//0033-2909.112.1.155). This information has been added to the revised manuscript. See below for details.

Author Action:

“*Due to the large sample size (and the increased likelihood of rejecting the null hypothesis when the null hypothesis is true), the adjusted R^2^ value had to increase by 1% or more in order for the predictor to be considered significant.* ***An R^2^ value of 1% represents the threshold for a small effect [38]****.*” (lines 275-276).

2. An alternate modelling approach is to perform a disaggregated analysis by subgroup and sex. This would bring the sample sizes down considerably, yet still provide a sufficient sample for the regression analyses (according to the sample sizes for the sex/subgroup cells shown in the S3 supplement). Perhaps this would enable use of more traditional statistical significance criteria (p-value versus the change in adjusted R2 threshold which is difficult to set)? Does this warrant consideration?

Author Response: We agree that a reasonable alternative modelling approach would involve a disaggregated analysis by both subgroup and sex. We did consider completing the analysis disaggregated by sex but opted to complete the analysis as it is currently presented for several reasons. Firstly, this analysis would have been performed if we observed a significant interaction effect (i.e. we would have completed post-hoc analyses for each subgroup). However, given that we did not observe a significant interaction effect, we did not complete the disaggregated analyses by subgroup. Secondly, disaggregating by subgroup would involve comparing the associations between individual subgroups rather than identifying associations across subgroups. This approach focuses on a different research question than what we wanted to investigate. Thirdly, given the number of subgroups and number of variables, a vast number of analyses would have been completed but not necessarily more informative or easily interpreted. Therefore, as an initial first step, the analyses were not disaggregated by sex. Perhaps however, future analyses could expand upon the multimorbidity subgroups.

Author Action: We have expanded the Limitations and Future Directions to address some of the limitations associated with the current analytic approach (i.e. subgroups not disaggregated).

*“****Limitations associated with the statistical modelling included the (i) definition of multimorbidity as a binary variable (presence or absence); (ii) inclusion of subgroup as a covariate rather than separate analyses per group; and (iii) criteria for statistical significance (R^2^ value increasing by 1% or more)****.* ***More sophisticated analyses that reflect the relative weight of different chronic health conditions and their association with CRP might yield different results, however there is no consensus on a list of chronic health conditions that are particularly associated with systemic inflammation.*** ***Additionally, distinguishing between the type of adipose tissue (e.g. visceral, subcutaneous, etc.) and its impact on inflammation would also be beneficial, although beyond the scope of the current study.****”* (lines 480-489).

*“Future directions might explore whether the proportion of body tissue attributed to fat might be a valuable clinical outcome for the conservative treatment of inflammatory chronic diseases such as OA. Additionally, exploring other markers of inflammation, such as interleukin-6 and tumour necrosis factor may be worthwhile to explore in OA to reflect different inflammatory pathways.* ***Finally, an analysis involving a greater number of subgroups that further partition chronic conditions may provide a better opportunity to identify the nuances associated with markers of inflammation and their role in obesity-related multimorbidities****.”* (lines 508-511).

4. Results:
i. Model Outcomes: Unstandardized and standardized betas (#’s, 95% CIs, figure) are provided in the main body of the manuscript for the linear regressions run for each of the eight dependent variables. It would be helpful to include the results from S5 in the main body of the manuscript. This explains the focus on the results (on only those achieving significance), and provides a sense of the statistical significance (or insignificance) and change in magnitude of the adjusted R2 across the eight outcomes.

Author Response: We agree that this table would be more helpful when presented with the main body of the manuscript. We have also added the unstandardized and standardized beta-coefficients for model 3 to the supplementary materal (even though the overall model was not significant).

Author Action: We have added the table from S5 to the main body of the manuscript (now referred to as Table 1), located between lines 306-307. The original Table 1 is now Table 2 in the main body of the manuscript.

The beta-coefficients for model 3 are now included in S4: *“The unstandardized and standardized β coefficients for log-CRP for model 2 (where log-CRP was added to the regression) are presented in Table* ***2~~1~~ and for model 3 (where log-CRP × subgroup interaction term was added to the regression) are presented in S4****.”* (lines 317-319).

ii. Additional Supplement: It would also be helpful to provide the full set of results for the linear regression models, that includes the results for the covariates. In particular, the subgroup results may be interesting to readers, as OA is discussed at length in the Introduction (and the subgroups are defined by the OA groups). This may also allow readers to assess the extent to which some of the hypothesized results emerged in the models (even if not significant according to the R2 threshold)– e.g., is CRP more strongly associated with physical function in those with lower extremity OA compared to hand OA?

Author Response: We agree that comparing results between different OA groups would be interesting. Unfortunately, because there was no significant interaction effect for any of the dependent variables, we did not present the results for each subgroup. Overall, in this study, the effect was uniform among subgroups. We also agree it may be informative for the reader to see the full set of results for the linear regression models that includes the covariates. Although we have presented the overall model for the covariates (i.e. adjusted R^2^ and associated *p-*value), we can also present the β-coefficients. We have added a table (S5) to the supplementary material that presents, as an example, the unstandardized and standardized β-coefficients and 95%CI for the covariates associated with DXA whole-body fat percent (which was significant for females and males). However, due to the overall number of covariates and dependent variables, it is not feasible to present the β-coefficients for all covariates for all models.

Author Action: The β-coefficients for covariates associated with one of the dependent variables has been added to S5: “**An example of the unstandardized and standardized β coefficients for covariates is presented in S5**.” (lines 318-319).

5. Discussion:
i. It is stated that the “lack of association of log-CRP with the number of multimorbidities as reflected by subgroup analyses was unexpected”, and that this contradicts findings in the literature relating CRP with inflammation and inflammation with many health conditions. This statement should be revised, because number of multimorbidities was not modelled directly in this study, it was captured in a subgroup where MM was defined as a binary variable. This modelling choice could account for the divergence of this study’s findings with the broader literature.

Author Response: We agree and thank you for highlighting this discrepancy. This sentence has been modified in the revised manuscript. See below for details.

Author Action:

*“The lack of association of log-CRP****, even among subgroups with multimorbidities, ~~with the number of multimorbidities as reflected by subgroup analyses~~*** *was unexpected because the level of CRP is proportional to the intensity of inflammation [****26****] and greater CRP levels are associated with many chronic health conditions [****18-25****]. However,* ***there are a number of reasons for this observation. First, multimorbidity was defined as a binary variable and was not modelled directly. Second,*** *there may exist considerable variability at any single point in time that results from a wide variety of multimorbidities. The time-course of some chronic conditions included in this analysis (e.g. rheumatoid arthritis) may not be steady, which may lead to fluctuating levels of CRP over time.* ***Third,*** ***self****-report may also confound the associations.”* (lines 449-458).

6. Limitations and Future Directions:
i. Limitations should be revised to include some of the modelling choices made in this study (e.g., MM defined as a binary variable, subgroup captured as a covariate versus disaggregation by subgroup) and criterion set for statistical significance (1%+ threshold for change in R2).

Author Response: We agree and these limitations have been added to the revised manuscript. See below for details.

Author Action:
*“We analyzed data previously collected through the CLSA. Although the CLSA is transparent with their data collection methods, we were not able to directly collect the data and thus, are somewhat removed from verifying the accuracy.* ***Limitations associated with the statistical modelling included the (i) definition of multimorbidity as a binary variable (presence or absence); (ii) inclusion of subgroup as a covariate rather than separate analyses per group; and (iii) criteria for statistical significance (R^2^ value increasing by 1% or more)****.* ***More sophisticated analyses that reflect the relative weight of different chronic health conditions and their association with CRP might yield different results, however there is no consensus on a list of chronic health conditions that are particularly associated with systemic inflammation. Additionally, distinguishing between the type of adipose tissue (e.g. visceral, subcutaneous, etc.) and its impact on inflammation would also be beneficial, although beyond the scope of the current study.****”* (lines 480-489).

7. Conclusion:
i. The second sentence states that the relationship is consistent “regardless of the number of chronic conditions”. This should be rephrased to state that the relationship is consistent” regardless of MM status” (number of chronic conditions was not captured in the analysis, only MM defined as a binary variable).

Author Response: We agree and this sentence has been modified.

Author Action: The sentence now reads as follows in the revised manuscript: *“This relationship is consistent regardless of* ***~~the number of chronic conditions~~ multimorbidity status****, sex and the presence of OA.”* (line 516).

Reviewer #2: In this manuscript, the authors seek to understand associations between serum inflammation (as measured via C-reactive protein, body composition, and physical capacity among Canadian adults with and without Osteoarthritis and/or multimorbidity. Following their analyses, they find that CRP levels are associated with body composition, regardless of OA or MM status, and some sex differences can also be observed. The paper is well written and very detailed. Additionally, it benefits from using a large accessible data set and study that used gold-standard methods for assessment of body composition (DXA). However, there are several revisions requested before the paper can move forward.

Author Response/Action: Thank you for these comments and the thoughtful review of our manuscript. We believe the manuscript is improved after responding to your comments.

1) It is recommended that greater detail and transparency be presented in the results and discussion regarding the associations measured between CRP and the subgroups, to better integrate the sections of the paper. There is a large focus on the associations between inflammation and OA, and multimorbidity in the introduction of the paper. However, these results get somewhat lost in the results section. The authors acknowledge they are not statistically significant, but without being able to view the models that include the subgroups, one cannot adjudicate whether they may indicate some degree of biological significance. Additionally, as the discussion delves into the observations that associations between degrees/types of OA and multimorbidity were negligible or absent, this should be more apparent in the results tables and text. The paper would also benefit from greater discussion regarding the potential reasons why the results here don't necessary reflect what has been found elsewhere.

Author Response: We agree that greater transparency regarding presentation of the results would benefit the overall discussion. As such, we have moved some of the tables in the supplementary files to the main document. See below for details.

Author Action: S5 has been moved to the main body and renamed Table 1. S4 has been moved to the main body and renamed Fig 2. A new S4 has been created and presents the unstandardized and standardized beta-coefficients for model 3.

2) The paper would benefit from removing some of the tables from the supporting information section, and moving them to the results. As presented, the results are difficult to follow given that many of the results are presented in the supporting information section.

Author Response: We agree and have moved some of the tables from the supplementary information to the main body of the manuscript. These tables provide the details of results that show the associations of CRP with dependent variables, disaggregated by sex. We have also added the beta-coefficients corresponding to log-CRP for model 3 (which includes covariates + log CRP + interaction term) where previously, we only included the unstandardized and standardized beta-coefficients for log-CRP pertaining to model 2 (which includes covariates + log CRP). See below for details.

Author Action: S5 has been moved to the main body and renamed Table 1. S4 has been moved to the main body and renamed Fig 2. A new S4 has been created and presents the unstandardized and standardized beta-coefficients for model 3.

3) As stated, the strongest associations were between central adiposity and CRP. Greater discussion of what may be causing that would benefit the paper. For example, does that association indicate that visceral adiposity (known to be strongly associated with pro-inflammatory cytokines) is contributing to these observations?

Author Response: We agree that greater discussion surrounding this point is warranted. We have revised the Discussion. See below for details.

Author Action:

*“In this large, cross-sectional analysis, central adiposity but not OA disease* ***~~(~~****or multimorbidity****~~)~~*** ***were~~as~~*** *related with systemic inflammation.* ***This greater central adiposity likely reflects greater visceral adiposity which is directly associated with increased cardiovascular and diabetes risk [40]. This greater risk for chronic cardiometabolic conditions could be explained by~~The~~ our observation that*** *accumulation of fatty tissue in the whole body and trunk was linked to elevated serum CRP.”* (lines 409-414).

4) the paper may also benefit from including additional future directions, such as recommendations for different types of analyses or ways to investigate that may better assess the relationship between adiposity, inflammation, and OA/multimorbidity.

Author Response: We agree and have added to the Future Directions section. See below for details.

Author Action:

*“Future directions might explore whether the proportion of body tissue attributed to fat might be a valuable clinical outcome for the conservative treatment of inflammatory chronic diseases such as OA. Additionally, exploring other markers of inflammation, such as interleukin-6 and tumour necrosis factor may be worthwhile to explore in OA to reflect different inflammatory pathways.* ***Finally, an analysis involving a greater number of subgroups that further partition chronic conditions may provide a better opportunity to identify the nuances associated with markers of inflammation and their role in obesity-related multimorbidities.****”* (lines 504-511).

The following minor revisions are also requested.
Page 3 Line 57 – 58: The phrasing is a bit redundant. Meaningful engagement is loaded/subjective and may need defining. Otherwise, I recommend replacing meaningful with 'typical'.

Author Response: This sentence has been revised. See below for details.

Author Action: The sentence now reads as follows: *“Ultimately, regardless of which joints are affected, OA is associated with reduced physical function [6] and physical activity [3] which challenges* ***~~meaningful~~typical*** *engagement in life.”* (line 60).

Page 3, line 61: add ‘and’ before altered

Author Response: This sentence has been revised. See below for details.

Author Action: The sentence now reads as follows: *“There are multiple mechanisms by which obesity contributes to OA, including abnormal biomechanics [8,9],* ***and*** *altered body composition and systemic inflammation [10-12].”* (line 63).

Page 3, line 65 – 67: This is a general; it would benefit from specificity (e.g., give examples of which pro-inflammatory cytokines).

Author Response: This sentence has been revised. See below for details.

Author Action: This sentence now reads as follows: *“The deposition of adipocytes produces inflammatory adipokines, such as increased leptin****, interleukin-6 and tumor necrosis factor-alpha as well as*** ***~~and~~*** *decreased adiponectin, which are associated with inflammatory biomarkers that contribute to chronic low-grade inflammation in obesity [13,14].”* (lines 67-68).

Page 4, line 87: comma after hand OA

Author Response/Action: A comma has been added after hand OA (line 102).

Page 4, line 88 – 89: better is subjective- replace with the specifics, (e.g., lower body fat %, lower BMI, etc.), and 'improved physical capacity"

Author Response: This sentence has been revised to address both the current comment as well as the next comment. See below for details.

Author Action: This sentence now reads as follows: *“We hypothesized that* ***~~lower~~higher*** *levels of CRP would be associated with* ***~~better body composition (lower~~greater*** *adiposity and* ***~~greater~~reduced*** *lean mass****~~)~~*** *and* ***poorer*** *physical capacity (****~~faster~~slower*** *walk time, chair rise time, standing balance and* ***~~greater~~reduced*** *grip strength).”* (lines 103-105).

Page 4, line 90: This sentence needs clarification. Following the prior sentence, the assumption is made that the hypothesis is that lower levels of CRP have a stronger association with OA compared to those without OA, etc.

Author Response/Action: Thank you for highlighting this discrepancy. To help clarify this sentence, we have modified the sentence prior (lines 103-105) so that the ‘direction’ of the hypothesis is consistent (i.e. both sentences now reflect worsening/poorer outcomes).

Page 5, line 114-115: Evaluate the tense of access here

Author Response: We agree and have revised this sentence. See below for details.

Author Action:

*“Further, this research team* ***~~accesses~~accessed*** *information of how data were collected from CLSA documentation.”* (line 131).

Page 7, line 162: is this hsCRP?

Author Response/Action: Thank you for highlighting the need for clarification of this sentence. At this point, we refer to “circulating CRP” instead of “hsCRP” because the circulating CRP has not yet been observed using a high-sensitivity ELISA method. Once the circulating CRP was measured using the high-sensitivity ELISA, it was referred to thereafter as “hs-CRP”.

Page 13, line 287 – 289: While the explanations are great, some of the detail isn't necessary. You should assume your reader will know that a CI that doesn't cross 0 is significant.

Author Response: We have removed this sentence. See below for details.

Author Action:

*“****~~The confidence intervals for each β coefficient for all dependent variables did not cross zero, indicating statistical significance. However, as noted earlier, the~~ The*** *magnitudes of change in the explained variance were small for 5 of the 8 dependent variables (indicated by an increase in the adjusted R^2^ by less than 1%) and therefore, the β coefficients for these dependent variables were not considered important.”* (lines 321-323).

Page 16: Results: As the paper is focused heavily on OA, there should be greater inclusion of results regarding that variable.

Author Response: We agree and have added more detail regarding the distribution of participants with OA. See below for details.

Author Action: We have added the percentage of participants with lower extremity OA, hand OA and multimorbidity for females and males to the Results section: “***The percentage of females with lower extremity OA, hand OA and multimorbidity was 22.4%, 16.6% and 46.4%, respectively. The percentage of males with lower extremity OA, hand OA and multimorbidity was 15.7%, 7.7% and 45.0%, respectively.***” (lines 288-291).

Page 17, lines 372 – 374: There needs to be greater delineation in the results section of how these levels of systemic inflammation are occurring regardless of OA or multimorbidity.

Author Response: We agree and have added more detail regarding the distribution of CRP values across sex and subgroups. See below for details.

Author Action: We have added the mean (SD) for CRP values for each sex and subgroup to the Results section: “***For females, the mean and standard deviation (SD) of CRP values for each subgroup (A to H) are 2.24 (4.54), 3.14 (5.73), 2.72 (4.79), 3.70 (5.60), 2.15 (5.21), 2.82 (3.85), 2.56 (3.45) and 4.01 (7.95), respectively. For males, the mean (SD) of CRP values for each subgroup (A to H) are 1.96 (4.39), 2.57 (4.99), 1.95 (3.78), 3.06 (5.43), 2.24 (5.12), 2.55 (5.14), 2.44 (4.55) and 4.12 (8.34), respectively.***” (lines 296-300).

Page 18, line 387 – 388: It may be beneficial to also discuss types of fat measured (e.g., subcutaneous vs. visceral) and how that may impact inflammation levels.

Author Response: We agree that distinguishing between the types of fat would provide greater insight. However, the data for all types of fat was not available for this analysis. As such, we had added this limitation to the Limitations section in the revised manuscript. See below for details.

Author Action:

“***Additionally, distinguishing between the type of adipose tissue (e.g. visceral, subcutaneous, etc.) and its impact on inflammation would also be beneficial, although beyond the scope of the current study.***” (lines 487-489).

Page 18, lines 391- 394: This isn't the focus of the paper; associations between CRP and more severe OA are not clear in the results presented.

Author Response/Action: We agree and have removed 1 sentence in the revised manuscript (lines 431-433).

Page 19, line 429: As the paper isn't assessing sarcopenic changes, this is speculative.

Author Response: The term “sarcopenic” has been removed. See details below.

Author Action: *“However, for males, there was a relationship between log-CRP and DXA appendicular lean mass index, suggesting sex-related differences in appendicular* ***~~sarcopenic~~lean mass*** *changes.”* (line 468).

Page 20, lines 446 – 447: As CRP increases with acute infection, was this controlled for?

Author Response/Action: Acute infection was not measured or controlled for.
