# Supplementary material for: Associations of body fat percentage with C-reactive protein levels in Canadian adults with and without osteoarthritis: Findings from the Canadian Longitudinal Study on Aging (CLSA)
Source: PLoS One. 2026 Feb 26;21(2):e0341604. doi: 10.1371/journal.pone.0341604 (PMC12944775; doi:10.1371/journal.pone.0341604)
Supplement: Attachment — Submitted filename: Review comments on associations with CRP.docx [file pone.0341604.s005.docx]

This study aimed to determine the cross-sectional associations of serum inflammation (CRP) with body composition and physical capacity in Canadian middle-aged and older adults with and without lower extremity OA, hand OA, and multimorbidities. The manuscript addresses an important and timely topic, particularly given the increasing prevalence of osteoarthritis and multimorbidity in aging populations. The focus on both body composition and physical capacity adds a valuable perspective to the existing literature, and the data analysis appears rigorous. I found the study to be highly interesting and relevant. However, I have a few suggestions for improvement and clarification, outlined below, that may help strengthen the clarity and impact of the manuscript.

**<Major points>**

In the first round, the authors focused primarily on addressing reviewer comments in a piecemeal manner. However, it is necessary to thoroughly re-evaluate the entire manuscript and revise it to ensure a coherent and logical flow throughout. Additionally, by only correcting the specific sections pointed out in round 1, the issues identified by the reviewers continue to appear in other parts of the manuscript.

**Abstract**

**1. Page 51, Line 39-40**: In the abstract, the phrase *“an increase in the adjusted R² between models by 1% or more was considered significant”* could be misleading, as the term *“significant”* is commonly associated with statistical significance (i.e., based on p-values). However, in this context, the 1% threshold represents a predefined criterion for practical or substantive improvement in model fit, not the result of a formal statistical test. To avoid potential misinterpretation, I suggest rephrasing this sentence to clarify that the threshold reflects a meaningful change in explanatory power rather than statistical significance.

**2. Line 41**: In the results, the term *“elevated CRP”* is used to describe the association between CRP and various body composition and physical capacity outcomes. However, based on the methods section, CRP was treated as a continuous variable and log-transformed for regression analyses. The term *“elevated”* may imply a dichotomous classification (e.g., high vs. normal), which could be misleading. To enhance clarity and accurately reflect the statistical approach, I recommend revising this to *“higher CRP levels”* or *“log-transformed CRP”* throughout the manuscript where appropriate.

**Introduction**

**1. Line 90-92**: The sentence beginning with "However, the associations between CRP with measures of physical function, mobility and physical activity in OA are unknown" seems somewhat disconnected from the preceding points. Consider relocating this sentence to follow the fourth point or revising the transition phrase (e.g., "Moreover" or "In addition") to improve the logical flow and clarify that this is an additional limitation rather than a direct contrast to previous points.

**2. Line 102-105**: The repeated use of “and” in this sentence makes the structure somewhat difficult to follow. I recommend rephrasing to clearly delineate the three main components of the hypothesis: (i) greater adiposity, (ii) reduced lean mass, and (iii) poorer physical capacity.

**3. Line 101:** The age range of the study participants is 45-85 years, which includes middle-aged individuals as well as older adults. The use of the term "older adults" alone may thus be somewhat misleading or imprecise. Consider clarifying or adjusting the terminology to "middle-aged and older adults" or explicitly stating the age range (e.g., "adults aged 45-85 years") to better reflect the participant demographic.

**Methods**

**1. Line 147-149**: Hand OA is often associated with OA in other joints, such as the knee and hip. Previous epidemiological studies have consistently reported an increased likelihood of multi-joint involvement among individuals with hand OA. Given this, how were participants with hand OA and concurrent OA in other joints handled in the analyses?

While Figure 1 provides some insight into how participants with both hand OA and other types of OA were categorized, this is not clearly described in the Methods section. Please clarify this in the Methods.

**2. Line 217**: The phrase “whole-body fat mass calculated by the DXA software using fat mass and weight” may be slightly confusing, as fat mass is typically a direct output of the DXA scan. You might consider rephrasing for clarity.

**3. Line 255**: The statement "The independent variable (CRP) was log-transformed" lacks an explanation. To enhance clarity and transparency, please briefly state the reason for this transformation (e.g., skewed distribution of CRP values).

**Results**

**1. Line 288-291**: Is the information described here presented in a table or figure?

**2. Line 295-296**: The current caption refers to the distribution of CRP values "across the 8 subgroups," but Figure 2 only presents distributions by sex (female vs. male), without subgroup stratification. Please revise the caption to accurately reflect the content of the figure.

**3. Line 313, 333**: The phrase “no significant change” in reference to the adjusted R² values may lead to confusion, as the term “significant” is often interpreted in a statistical sense (i.e., p-value < 0.05). However, in this context, it appears to reflect a predefined threshold for a meaningful increase in explained variance (≥1%). I suggest rephrasing this to avoid ambiguity, perhaps using “no meaningful increase” or “no substantial change in explained variance”, and clarifying that this is based on effect size rather than statistical significance. Such ambiguous expressions are used throughout the manuscript, and I recommend reviewing the text carefully to identify and revise them accordingly.

**4. Line 333-335**: Where can the data corresponding to this description be accessed?

**5. Line 341-342**: The phrase “significantly different” could be interpreted in multiple ways—whether referring to the statistical significance of the overall model comparison (e.g., Model 1 vs. Model 2), or to the individual significance of the log-CRP term added in Model 2. Please clarify this.

This type of expression appears repeatedly throughout the subsequent results as well, so please revise accordingly.

**Discussion**

**1. Line 393, 407**: Please ensure that the description of participant age is stated accurately here as well.

**2. Line 477**: As the current study is cross-sectional in nature, causal relationships between CRP and the various physical and body composition outcomes cannot be inferred. However, this limitation is not clearly acknowledged in the manuscript. I recommend explicitly stating this as a limitation, to prevent potential overinterpretation of the observed associations.

**<Minor points>**

**Methods**

**1. Line 213**: Although the full term "Timed Up and Go" is mentioned earlier in the text, the corresponding abbreviation "tug" is not introduced in parentheses at that point. The abbreviation later appears without prior clarification, which may confuse readers. To improve clarity and maintain consistency, please introduce the abbreviation in parentheses when the term is first mentioned (i.e., "Timed Up and Go (TUG)").
