# Supplementary material for: Associations of body fat percentage with C-reactive protein levels in Canadian adults with and without osteoarthritis: Findings from the Canadian Longitudinal Study on Aging (CLSA)
Source: PLoS One. 2026 Feb 26;21(2):e0341604. doi: 10.1371/journal.pone.0341604 (PMC12944775; doi:10.1371/journal.pone.0341604)
Supplement: Attachment — Submitted filename: Review.docx [file pone.0341604.s006.docx]

**Title and Abstract**

**Strengthen the title to emphasize the main finding**

- Suggest:
  **"Associations of Body Fat Percentage with C Reactive Protein Levels in Older Canadians With and Without Osteoarthritis: Findings from the Canadian Longitudinal Study on Aging"**

**Clarify objective in the abstract**

- Add more explicit statement about *the strength of association* and *lack of OA specific effect* in the conclusion.

**Suggested Abstract Conclusion revision:**
"Percent adiposity but not osteoarthritis status was consistently associated with systemic inflammation, suggesting that adiposity driven inflammation may contribute to OA related health outcomes."

**Introduction**

**Clarify hypothesis formulation**

- Currently, the hypotheses about interactions with OA status and sex are a bit broad. Clarify that the study is exploratory regarding interaction terms but was powered to test main effects.

**Strengthen rationale for including multimorbidity**

- Briefly explain why multimorbidity is important in the OA inflammation link. E.g., "Given the frequent co-occurrence of chronic conditions in aging, understanding whether inflammation is uniquely driven by OA or shared with other comorbidities is critical."

**Methods**

**Participant Selection**

- Provide flow chart or explicit mention of numbers excluded at each step (CONSORT-style).

**Statistical Analysis**

- Explicitly mention how missing data were handled.
  - If excluded case-wise: "Analyses were conducted using complete case analysis; no imputation was performed."

**Effect size criteria**

- Clarify that "adjusted R² increases ≥1%" were used as thresholds for practical significance, in addition to p-values corrected for multiple comparisons.

**Interaction terms**

- Explain why OA subgroup interaction terms did not add value to model interpretation.

**Results**

**Table 1**

- Move all results tables (Table 1, S5) into a main results file or Supplementary File with clear captions.
- Summarize the main effects and emphasize effect sizes in the text.

**Figures**

- Ensure Figure 2 has clear axis labels, legend, and a description in the Results.
- Possibly combine or simplify figures to enhance clarity.

**Sex differences**

- Highlight key differences between females and males consistently across sections.

**Strengthen Discussion of Clinical Relevance**

- Relate the finding of small effect sizes (β ~0.13–0.16) to potential clinical impact on OA progression and management.
- Discuss limitations of CRP as a single inflammatory marker and suggest future studies exploring other biomarkers like IL-6, TNF-α.

**Multimorbidity discussion**

- Expand why the interaction term with multimorbidity did not yield significant changes in variance explained.

**Limitations**

- Include an explicit note on generalizability given the demographic profile (Canadian older adults) and the self-reported nature of OA diagnosis.

**Financial Disclosure**

**Clarify that funders had no role**

- Suggested sentence:
  "The funders had no role in study design, data collection and analysis, decision to publish, or preparation of the manuscript."

**Add URLs of funders**

- E.g.:
  - CIHR: https://cihr-irsc.gc.ca
  - Arthritis Society: <https://arthritis.ca>

**Data Availability**

**State de-identified data are available**

- Suggested statement:
  "Data are available from the Canadian Longitudinal Study on Aging ([www.clsa-elcv.ca](http://www.clsa-elcv.ca)) for researchers who meet the criteria for access to de-identified CLSA data."

**Technical Edits**

**Check line numbers**

- Some lines exceed 2-3 lines per sentence, consider splitting for readability.

**Abbreviations**

- Define all abbreviations on first use (e.g., OA, CRP, DXA).

**Reference formatting**

- Ensure references follow PLOS ONE’s citation style consistently.
