# Supplementary material for: Associations of body fat percentage with C-reactive protein levels in Canadian adults with and without osteoarthritis: Findings from the Canadian Longitudinal Study on Aging (CLSA)
Source: PLoS One. 2026 Feb 26;21(2):e0341604. doi: 10.1371/journal.pone.0341604 (PMC12944775; doi:10.1371/journal.pone.0341604)
Supplement: Attachment — Submitted filename: Response_to_Reviewers_PLOS_One_auresp_2.docx [file pone.0341604.s007.docx]

All tracked changes and revisions are provided in the document “Revised Manuscript With Tracked Changes_PLOS One”. Changes are provided in **bolded red font**. In the current document, original reviewer comments are provided in **blue** font and the response to the reviewer comments are provided in **black** font.

**Reviewer 1**

Title and Abstract

Strengthen the title to emphasize the main finding

- Suggest:
  "Associations of Body Fat Percentage with C Reactive Protein Levels in Older Canadians With and Without Osteoarthritis: Findings from the Canadian Longitudinal Study on Aging"

Author Response: Thank you for taking the time to review the manuscript in detail. We agree with this suggestion and have made the following changes.

Author Action: The title has now been changed to *“Associations of body fat percentage with C-reactive protein levels in Canadian adults with and without osteoarthritis: Findings from the Canadian Longitudinal Study on Aging (CLSA)”* (lines 1-3).

Clarify objective in the abstract

- Add more explicit statement about *the strength of association* and *lack of OA specific effect* in the conclusion.
- Suggested Abstract Conclusion revision:
  "Percent adiposity but not osteoarthritis status was consistently associated with systemic inflammation, suggesting that adiposity driven inflammation may contribute to OA related health outcomes."

Author Response: Thank you for this suggestion. We have made the following changes.

Author Action: The Conclusion in the Abstract now reads as follows *“**There were small but meaningful associations between CRP and each of percent adiposity and appendicular lean mass index. These associations were present regardless of OA and multimorbidity status. Percent adiposity, but not OA status, was consistently associated with systemic inflammation, suggesting that adiposity driven inflammation may contribute to OA related health outcomes.”* (lines 46-52).

Introduction

Clarify hypothesis formulation

- Currently, the hypotheses about interactions with OA status and sex are a bit broad. Clarify that the study is exploratory regarding interaction terms but was powered to test main effects.

Author Response: Thank you for this suggestion. We have made the following changes.

Author Action: The hypothesis (with revisions **bolded)** now reads as follows “*We hypothesized that higher levels of CRP would be associated with greater adiposity and reduced lean mass and poorer physical capacity (slower walk time, chair rise time, standing balance and reduced grip strength). We also hypothesized that these associations would be stronger in subgroups with OA compared to subgroups without OA; in females compared to males; and in those with knee or hip OA compared to hand OA.* ***Although this study is exploratory regarding interaction effects between OA status, multimorbidity status and sex, it was adequately powered to investigate the main effects.***” (lines 108-110).

Strengthen rationale for including multimorbidity

- Briefly explain why multimorbidity is important in the OA inflammation link. E.g., "Given the frequent co-occurrence of chronic conditions in aging, understanding whether inflammation is uniquely driven by OA or shared with other comorbidities is critical."

Author Response: Thank you for this suggestion. We have made the following changes.

Author Action: The purpose (with revisions **bolded**) now reads as follows “*The purpose of this study was to determine the cross-sectional associations of serum inflammation (CRP) with body composition and physical capacity in Canadian adults with and without lower extremity OA, hand OA, and multimorbidities.* ***Given the frequent co-occurrence of chronic conditions in aging, understanding whether inflammation is uniquely driven by OA or shared with other comorbidities is critical.***” (lines 101-103).

Methods

Participant Selection

- Provide flow chart or explicit mention of numbers excluded at each step (CONSORT-style).

Author Response: Thank you for this great suggestion. We have made the following changes.

Author Action: A new figure (now Figure 2) has been provided. This figure depicts the general inclusion/exclusion of participants based on availability of data as well as separation by sex (referenced lines 304-308 and 318).

Statistical Analysis

- Explicitly mention how missing data were handled.
  - If excluded case-wise: "Analyses were conducted using complete case analysis; no imputation was performed."

Author Response: Thank you for this suggestion. We have made the following changes.

Author Action: The following “***Analyses were conducted using complete case analysis; no imputation was performed.***” has been added to the Statistical Analysis (lines 300-301).

Effect size criteria

- Clarify that "adjusted R² increases ≥1%" were used as thresholds for practical significance, in addition to p-values corrected for multiple comparisons.

Author Response: Thank you for this suggestion. We have made the following changes.

Author Action: The following “***Overall, an adjusted R^2^ increase ≥1% were used as thresholds for practical and meaningful change, in addition to p-values corrected for multiple comparisons.***” has been added to the Statistical Analysis (lines 296-298).

Interaction terms

- Explain why OA subgroup interaction terms did not add value to model interpretation.

Author Response: Thank you for this suggestion. We have made the following changes.

Author Action: The following “***Therefore, the patterns observed in associations between CRP and measures of functional capacity and body composition were similar in females and males, with the single exception indicating that, in males, appendicular lean mass index was inversely associated with systemic inflammation. These associations were also not altered by variations in subgroup status; thus, we did not observe a stronger (or weaker) association of CRP in the presence of multimorbidities or OA.***” has been added to the Discussion (lines 458-463).

Results

Table 1

- Move all results tables (Table 1, S5) into a main results file or Supplementary File with clear captions.

Author Response: Thank you for this suggestion. We have made the following changes.

Author Action: We have moved Table 1 and Table 2 from the main document file to a new file titled “Main Tables_PLOS One”. We have also moved the tables from S4 and S5 to the new file (Main Tables_PLOS One) and renamed as appropriate.

- Summarize the main effects and emphasize effect sizes in the text.

Author Response: Thank you for this suggestion. As outlined (lines 347-350), there was no significant main effects for 5 of the 8 dependent variables. For the remaining 3 dependent variables (DXA whole body fat percent, DXA trunk fat percent, DXA appendicular lean mass index), there were significant main effects and details regarding these main effects are provided further down. However, to direct the reader towards these significant main effects, we have made the following changes.

Author Action: The following sentence “***Detailed results for the remaining 3 dependent variables (DXA whole body fat percent, DXA trunk fat percent, DXA appendicular lean mass index) are presented below.***” has been added to the Model Outcomes (lines 350-352) to help direct readers to the significant main effects for 3 of the 8 dependent variables.

Figures

- Ensure Figure 2 has clear axis labels, legend, and a description in the Results.

Author Response: Thank you for this suggestion. We have made the following changes.

Author Action: A sentence briefly describing Figure 2 (renamed Figure 3) is presented between lines 308-309. The description for the new Figure 3 is presented between lines 333-335.

- Possibly combine or simplify figures to enhance clarity.

Author Response: Thank you for this suggestion. We have made the following changes.

Author Action: Four tables (Table 1, Table 2, S4, S5) have been consolidated to one file “Main Tables_PLOS One”. This has reduced the supplementary file number from 5 to 3 figures/tables. We have also carefully reviewed figure contents to enhance simplicity.

Sex differences

- Highlight key differences between females and males consistently across sections.

Author Response: Thank you for this suggestion. We have made the following changes.

Author Action: The following sentence “***Therefore, the patterns observed in associations between CRP and measures of functional capacity and body composition were similar in females and males, with the single exception indicating that, in males, appendicular lean mass index was inversely associated with systemic inflammation.***” has been added to the Discussion (lines 458-461).

Strengthen Discussion of Clinical Relevance

- Relate the finding of small effect sizes (β ~0.13–0.16) to potential clinical impact on OA progression and management.

Author Response: Thank you for this suggestion. We have made the following changes.

Author Action: The following sentence “***However, it should be noted that the effect size is small (Table 2) and this likely indicates that the influence of systemic inflammation on the development and progression of OA is only a small part of a complex system of interconnected factors.***” has been added to the first paragraph of the Discussion (lines 470-472).

- Discuss limitations of CRP as a single inflammatory marker and suggest future studies exploring other biomarkers like IL-6, TNF-α.

Author Response: Thank you for this suggestion. We have made the following changes.

Author Action: The following sentence “***CRP is regulated by other pro-inflammatory cytokines, including interleukin-6 and tumor necrosis factor-alpha, which are secreted by various inflammatory and immunological cells [52]. Future studies would benefit from exploring these pro-inflammatory mediators to better determine the mechanisms underlying the association between OA, multimorbidity, body composition and physical capacity.***” has been added to the Discussion (lines 519-523).

Multimorbidity discussion

- Expand why the interaction term with multimorbidity did not yield significant changes in variance explained.

Author Response: Thank you for this suggestion. We have made the following changes.

Author Action: The following sentences “*First, multimorbidity was defined as a binary variable* ***and derived from a large variety of chronic conditions****.*” (lines 514-516) and “*Third,* ***subgroup as a variable, was defined according to a combination of hand OA, lower extremity OA and multimorbidity status. We were particularly interested in the interaction between CRP and subgroup. As a result however, the association with multimorbidity was not directly measured.*** *Fourth,…*” (lines 523-527) have been added to the Discussion.

Limitations

- Include an explicit note on generalizability given the demographic profile (Canadian older adults) and the self-reported nature of OA diagnosis.

Author Response: Thank you for this suggestion. We have made the following changes.

Author Action: The following sentences “***Additionally, the CLSA is limited to Canadian adults aged 45-85 years. Therefore, the generalizability of the dataset and any results derived from such data is limited to similar populations.***” have been added to the Discussion (lines 550-552).

Financial Disclosure

Clarify that funders had no role

- Suggested sentence:
  "The funders had no role in study design, data collection and analysis, decision to publish, or preparation of the manuscript."

Author Response: Thank you for noticing that this statement was missing from the main body of the manuscript.

Author Action: The funding information has been added to the main body of the manuscript. We have added the following “***The funders had no role in study design, data collection and analysis, decision to publish, or preparation of the manuscript.***” to the funding information on lines 615-617.

Add URLs of funders

- E.g.:
  - CIHR: https://cihr-irsc.gc.ca
  - Arthritis Society: [https://arthritis.ca](https://arthritis.ca)

Author Response: Thank you for this suggestion. We have made the following changes.

Author Action: We have added the URL for the funders (CIHR and The Arthritis Society). The funding has been added to the main body of the manuscript on lines 610 and 613.

Data Availability

State de-identified data are available

- Suggested statement:
  "Data are available from the Canadian Longitudinal Study on Aging ([www.clsa-elcv.ca](http://www.clsa-elcv.ca)) for researchers who meet the criteria for access to de-identified CLSA data."

Author Response: Thank you for this suggestion.

Author Action: We have modified the current statement to clarify that deidentified data is available. The Data Availability statement now reads as follows “***De-identified*** *data are available from the Canadian Longitudinal Study on Aging (www.clsa-elcv.ca) for researchers who meet the criteria for access to de-identified CLSA data. Inquiries regarding data access and availability can be made via email (*[*info@clsa-elcv.ca*](mailto:info@clsa-elcv.ca)*) and online (*[*https://www.clsa-elcv.ca/data-access/)*](https://www.clsa-elcv.ca/data-access/))*.*” (lines 620-623).

Technical Edits

Check line numbers

- Some lines exceed 2-3 lines per sentence, consider splitting for readability.

Author Response: Thank you for this suggestion.

Author Action: We have revised the following:

Lines 71-75: “*The deposition of adipocytes produces inflammatory adipokines, such as increased leptin, interleukin-6 and tumor necrosis factor-alpha as well as decreased adiponectin, which are associated with inflammatory biomarkers.* ***These changes*** ***~~that~~*** *contribute to chronic low-grade inflammation in obesity [13,14].*”

Lines 479-482: “*In general, fat mass percentage is higher in individuals with OA compared to controls [41].* ***~~and t~~****This adiposity likely worsens OA disease through multiple mechanisms, such as insulin resistance, altered immune response and inflammatory cells, degradation of cartilage [10] and reduced physical performance in those with OA [42].*”

Lines 502-507: “*It should be noted that previous analyses included participants that were in good physical and mental health [48], excluded participants using anabolic steroids and/or corticosteroids [47] and excluded participants who had higher CRP levels (>10 mg/L)* ***[49]****.* ***Those with higher CRP levels who were excluded*** ***~~who~~*** *were* ***~~also~~*** *more likely to have lower education, currently smoking, hypertensive, less physically active and report greater limitations with activities of daily living [49].*”

Abbreviations

- Define all abbreviations on first use (e.g., OA, CRP, DXA).

Author Response: Thank you for identifying any discrepancies in defining the abbreviations.

Author Action: We have defined DXA on its first use on lines 230-231. The sentence now reads as follows “*The following variables were acquired:* ***Dual-Energy X-Ray Absorptiometry*** *(DXA)* ***whole-body fat mass (g) (calculated using*** *fat mass and weight)…*”

Reference formatting

- Ensure references follow PLOS ONE’s citation style consistently.

Author Response: Thank you for this suggestion.

Author Action: We have reviewed PLOS ONE’s citation style criteria and compared with the current reference list. We have marked any necessary changes in the References section.

**Reviewer 2**

This study aimed to determine the cross-sectional associations of serum inflammation (CRP) with body composition and physical capacity in Canadian middle-aged and older adults with and without lower extremity OA, hand OA, and multimorbidities. The manuscript addresses an important and timely topic, particularly given the increasing prevalence of osteoarthritis and multimorbidity in aging populations. The focus on both body composition and physical capacity adds a valuable perspective to the existing literature, and the data analysis appears rigorous. I found the study to be highly interesting and relevant. However, I have a few suggestions for improvement and clarification, outlined below, that may help strengthen the clarity and impact of the manuscript.

Author Response: Thank you for this positive response and taking the time to review in detail. Answers and revisions to specific comments are addressed below.

Author Action: None.

<Major points>

In the first round, the authors focused primarily on addressing reviewer comments in a piecemeal manner. However, it is necessary to thoroughly re-evaluate the entire manuscript and revise it to ensure a coherent and logical flow throughout. Additionally, by only correcting the specific sections pointed out in round 1, the issues identified by the reviewers continue to appear in other parts of the manuscript.

Author Response: Thank you for this comment. We have carefully read the manuscript for readability and made editorial changes to improve the logical flow of all sections of the manuscript. We have also carefully reviewed comments from Round 1 to apply changes throughout the manuscript.

Author Action: We have reviewed comments from Round 1 and ensured all elements of the manuscript consistently reflect the requested changes, with the exception of any change requested in this current review. For example, the first revision requested tables of data be moved from supplementary to the main body of the manuscript; but this current review suggests the opposite. We have stayed consistent with the requests of the current review in these instances.

Abstract

1. Page 51, Line 39-40: In the abstract, the phrase *“an increase in the adjusted R² between models by 1% or more was considered significant”* could be misleading, as the term *“significant”* is commonly associated with statistical significance (i.e., based on p-values). However, in this context, the 1% threshold represents a predefined criterion for practical or substantive improvement in model fit, not the result of a formal statistical test. To avoid potential misinterpretation, I suggest rephrasing this sentence to clarify that the threshold reflects a meaningful change in explanatory power rather than statistical significance.

Author Response: Thank for highlighting this important difference. We agree and have made the following revisions where appropriate.

Author Action: The following revisions were completed.

Line 41: “*A priori, the research team defined that an increase in the adjusted R^2^ between models by 1% or more was considered* ***a meaningful change in explanatory power ~~significant~~****.*”

Line 287-289: “*Due to the large sample size (and the increased likelihood of rejecting the null hypothesis when the null hypothesis is true), the adjusted R^2^ value had to increase by 1% or more in order for the predictor to be considered* ***~~significant~~ a meaningful change in explanatory power.***”

Lines 296-298: “***Overall, an adjusted R^2^ increase ≥1% were used as thresholds for practical and meaningful change, in addition to p-values corrected for multiple comparisons.***”

Line 345: “*Model 1 (covariates only) explained* ***a meaningful amount of the*** *variance for all dependent variables, in both females and males.*”

Line 348: “*…no* ***~~significant~~ meaningful*** *change…*”

Line 378: “In model 3, for each of the dependent variables, **no meaningful** change…”

Lines 385-386: “***When log-CRP was added to model 1, there was a meaningful increase in the explained variance of the model*** *(model 2) (Table1).*”

Lines 392-394: “***When the*** *interaction term (log-CRP × subgroup)* ***was added to model 2, there was no meaningful change in the explained variance of the model (****model 3****)*** *(Table* ***2****)*.”

Lines 396-398: “***When log-CRP was added to model 1, there was a meaningful increase in the explained variance of the model (model 2)*** *(Table* ***2****).*”

Lines 403-405: “***When the*** *interaction term (log-CRP × subgroup)* ***was added to model 2, there was no meaningful change in the explained variance of the model (****model 3****)*** *(Table* ***2****)*.”

Lines 409-411: “***When log-CRP was added to model 1, there was a meaningful increase in the explained variance of the model (****model 2****)*** *(Table* ***2****).*”

Lines 415-418: “***When the*** *interaction term (log-CRP × subgroup)* ***was added to model 2, there was no meaningful change in the explained variance of the model (****model 3****)*** *(Table* ***2****).*”

Lines 420-422: “***When log-CRP was added to model 1, there was a meaningful increase in the explained variance of the model*** *(model 2) (Table* ***2****).*”

Lines 426-429: “***When the*** *interaction term (log-CRP × subgroup)* ***was added to model 2, there was no meaningful change in the explained variance of the model (****model 3****)*** *(Table* ***2****).*”

Line 432: “*For females,* ***there was no meaningful*** *change in the explained variance* ***of the model*** *(represented by an increase in the adjusted R^2^ by 1% or more)…*”

Lines 437-439: “***When log-CRP was added to model 1, there was a meaningful change in the explained variance of the model*** *(model 2)…*”

Lines 444-446: “***When the*** *interaction term (log-CRP × subgroup)* ***was added to model 2, there was no meaningful change in the explained variance of the model (****model 3****)*** *(Table* ***2****).*”

2. Line 41: In the results, the term *“elevated CRP”* is used to describe the association between CRP and various body composition and physical capacity outcomes. However, based on the methods section, CRP was treated as a continuous variable and log-transformed for regression analyses. The term *“elevated”* may imply a dichotomous classification (e.g., high vs. normal), which could be misleading. To enhance clarity and accurately reflect the statistical approach, I recommend revising this to *“higher CRP levels”* or *“log-transformed CRP”* throughout the manuscript where appropriate.

Author Response: Thank for highlighting this subtle difference. We agree and have made the following revisions where appropriate.

Author Action: The following lines have been revised from “elevated CRP” to a version of “higher CRP levels” where appropriate: line 42, line 79, line 85, line 90, line 97, line 478, line 484, line 495, line 531, lines 535-536.

Introduction

1. Line 90-92: The sentence beginning with "However, the associations between CRP with measures of physical function, mobility and physical activity in OA are unknown" seems somewhat disconnected from the preceding points. Consider relocating this sentence to follow the fourth point or revising the transition phrase (e.g., "Moreover" or "In addition") to improve the logical flow and clarify that this is an additional limitation rather than a direct contrast to previous points.

Author Response: Thank you for highlighting this need for clarification. The purpose of this point was to highlight that there is evidence suggesting an association between CRP with symptomatic and structural disease changes in OA but there lacks a similar association between CRP with mobility and physical function measures. We have made the following changes to hopefully communicate this idea better.

Author Action: The third point of the objective/hypothesis now reads as follows “*Third,* *in OA,* ***~~elevated~~ higher*** *CRP concentrations are linked* ***with symptomatic and structural disease changes, including*** *(i) worse pain [18-20], (ii) greater disease incidence [20] and (iii) faster disease progression based on radiography [21], although results are somewhat inconsistent between studies [18-21, 27].* ***Whether a similar association with higher CRP levels exists for ~~However, the associations between CRP with~~*** *measures of physical function, mobility and physical activity in OA are unknown.*” (lines 90-95).

2. Line 102-105: The repeated use of “and” in this sentence makes the structure somewhat difficult to follow. I recommend rephrasing to clearly delineate the three main components of the hypothesis: (i) greater adiposity, (ii) reduced lean mass, and (iii) poorer physical capacity.

Author Response: Thank you for this suggestion. We agree and have made the following changes.

Author Action: The sentence now reads as follows “*We hypothesized that higher levels of CRP would be associated with* ***(i)*** *greater adiposity,* ***(ii)*** *reduced lean mass, and* ***(iii)*** *poorer physical capacity…*” (lines 103-105).

3. Line 101: The age range of the study participants is 45-85 years, which includes middle-aged individuals as well as older adults. The use of the term "older adults" alone may thus be somewhat misleading or imprecise. Consider clarifying or adjusting the terminology to "middle-aged and older adults" or explicitly stating the age range (e.g., "adults aged 45-85 years") to better reflect the participant demographic.

Author Response: Thank you for this suggestion. We agree and have made the following changes.

Author Action: The term “older” has been removed and replaced with the specific age range (45-85 years) where appropriate: line 2, line 100, line 113, line 449, line 468, line 587.

Methods

1. Line 147-149: Hand OA is often associated with OA in other joints, such as the knee and hip. Previous epidemiological studies have consistently reported an increased likelihood of multi-joint involvement among individuals with hand OA. Given this, how were participants with hand OA and concurrent OA in other joints handled in the analyses?

Author Response: Thank you for this question. This is an important consideration and we agree, given previously published research. As per Figure 1, Subgroups G and H included participants that self-reported having both hand OA and lower extremity (knee, hip, both) OA. The only differentiation between subgroup G and H was multimorbidity status.

Author Action: We have provided further clarification of the subgroup designations by adding the following “***Participants in subgroup A self-reported none of these conditions and thus, subgroup A is a healthy reference group in this sample. In contrast, participants in subgroup H self-reported the presence of all three of these health conditions. The subgroups in between A and H self-reported various combinations of lower extremity OA, hand OA and multimorbidity. For example, individuals with both lower extremity OA and hand OA but differing multimorbidity status, are represented by subgroups G and H.***” (lines 172-177).

While Figure 1 provides some insight into how participants with both hand OA and other types of OA were categorized, this is not clearly described in the Methods section. Please clarify this in the Methods.

Author Response: Thank you for highlighting that this area of the manuscript requires greater clarification. A description of how lower extremity OA, hand OA and multimorbidity status was determined is presented from lines 146-177. However, we have revised this section in several places to help clarify the classification method.

Author Action: The following statements have been added to the revised manuscript:

Lines 151-152: “***Therefore, if a participant did not self-report a diagnosis of knee OA or hip OA, this participant was classified as having “no lower extremity OA” (Fig 1).***”

Lines 154-155: “***Therefore, if a participant did not self-report a diagnosis of hand OA, this participant was classified as having “no hand OA” (Fig 1).***”

Lines 168-170: “***Therefore, if a participant did not self-report a diagnosis 2 or more of the listed conditions, this participant was classified as having “no multimorbidity” (Fig 1).***”

Lines 172-177: “***Participants in subgroup A self-reported none of these conditions and thus, subgroup A is a healthy reference group in this sample. In contrast, participants in subgroup H self-reported the presence of all three of these health conditions. The subgroups in between A and H self-reported various combinations of lower extremity OA, hand OA and multimorbidity. For example, individuals with both lower extremity OA and hand OA but differing multimorbidity status, are represented by subgroups G and H.***”

2. Line 217: The phrase “whole-body fat mass calculated by the DXA software using fat mass and weight” may be slightly confusing, as fat mass is typically a direct output of the DXA scan. You might consider rephrasing for clarity.

Author Response: Thank you for this suggestion. We have revised the statement for clarity.

Author Action: The sentence now reads as follows “*The following variables were acquired:* ***Dual-Energy X-Ray Absorptiometry*** *(DXA)* ***whole-body fat mass (g) (calculated using*** *fat mass and weight)…*” (lines 230-232).

3. Line 255: The statement "The independent variable (CRP) was log-transformed" lacks an explanation. To enhance clarity and transparency, please briefly state the reason for this transformation (e.g., skewed distribution of CRP values).

Author Response: Thank you for this suggestion. We have revised the sentence as follows.

Author Action: The statement now reads as follows “*The independent variable (CRP) was log-transformed* ***because of a skewed distribution.***” (lines 269-270).

Results

1. Line 288-291: Is the information described here presented in a table or figure?

Author Response: Thank you for this comment. This information was not in a table or figure. However, this information is easier to read in a table format.

Author Action: We have moved the information from this section to a new Table 1 (lines 322-331). Subsequent tables have been renumbered.

2. Line 295-296: The current caption refers to the distribution of CRP values "across the 8 subgroups," but Figure 2 only presents distributions by sex (female vs. male), without subgroup stratification. Please revise the caption to accurately reflect the content of the figure.

Author Response: Thank you for this comment. You are correct that the original Figure 2 is not separated according to subgroup. The caption for Figure 2 (renamed Figure 3) currently reads as follows “*Violin plot showing the distribution of CRP values, separated by sex. A total of 541 (4.0%) females and 386 (2.9%) males had levels greater than 10mg/L, which in a clinical context, are indicative of clinically significant inflammation.*”. However, the section identified in the current comment refers to text within the manuscript that describes the mean (SD) of CRP values across the subgroups for females/males.

Author Action: As per a previous comment, this information has been moved to a new Table 1 (lines 322-331).

3. Line 313, 333: The phrase “no significant change” in reference to the adjusted R² values may lead to confusion, as the term “significant” is often interpreted in a statistical sense (i.e., p-value < 0.05). However, in this context, it appears to reflect a predefined threshold for a meaningful increase in explained variance (≥1%). I suggest rephrasing this to avoid ambiguity, perhaps using “no meaningful increase” or “no substantial change in explained variance”, and clarifying that this is based on effect size rather than statistical significance. Such ambiguous expressions are used throughout the manuscript, and I recommend reviewing the text carefully to identify and revise them accordingly.

Author Response: Thank you for highlighting this subtle difference in terminology. We have made the following changes.

Author Action: In several places throughout the revised manuscript as well as Table 2, the term “significant” was replaced with an appropriate version of “meaningful change”. We had also clarified that our predefined threshold of >1% was chosen to define a meaningful change in terms of effect size, rather than statistical significance (lines 41, line 289, lines 296-298, lines 575-577).

4. Line 333-335: Where can the data corresponding to this description be accessed?

Author Response: Thank you for highlighting this lack of information. We have made the following changes.

Author Action: Data pertaining to this statement is presented in Table 2 and 4. Therefore, we have added reference to table 2 and 4 within the revised manuscript. This statement now reads as follows “*In model 3, for each of the dependent variables, no meaningful change in the explained variance (represented by an increase in the adjusted R^2^ by 1% or more) was observed with the addition of the interaction term (log-CRP × subgroup) to the regression* ***(Table 2, Table 4).***” (lines 378-381).

5. Line 341-342: The phrase “significantly different” could be interpreted in multiple ways—whether referring to the statistical significance of the overall model comparison (e.g., Model 1 vs. Model 2), or to the individual significance of the log-CRP term added in Model 2. Please clarify this.

This type of expression appears repeatedly throughout the subsequent results as well, so please revise accordingly.

Author Response: Thank you for highlighting this discrepancy. As per previous comments, we have modified the term “significant” and rewritten this sentence (and similar sentences) to clarify that this phrase is referring to a change in the overall explained variance of the model when a new variable (either log-CRP for model 2 or an interaction term for model 3) is added to the existing model.

Author Action: In several places throughout the Results section, we have modified these sentences to clarify the comparison between models: lines 349, 386, 393, 396, 397, 404, 409, 410, 416, 417, 420, 421, 427, 428, 433, 437, 439, 445)

Discussion

1. Line 393, 407: Please ensure that the description of participant age is stated accurately here as well.

Author Response: Thank you for this suggestion. We have made the following changes.

Author Action: We have added the participant age as well as regional information (i.e. Canadians) in these two sections (lines 473, 488-489) as well as several other areas within the manuscript (lines 113, 449, 551).

2. Line 477: As the current study is cross-sectional in nature, causal relationships between CRP and the various physical and body composition outcomes cannot be inferred. However, this limitation is not clearly acknowledged in the manuscript. I recommend explicitly stating this as a limitation, to prevent potential overinterpretation of the observed associations.

Author Response: Thank you for highlighting this important limitation. We have made the following changes.

Author Action: We have added a sentence regarding the use of cross-sectional data that limits evaluation of a causal relationship. The limitations now include the following “*Limitations associated with the statistical modelling included the (i) definition of multimorbidity as a binary variable (presence or absence); (ii) inclusion of subgroup as a covariate rather than separate analyses per group; (iii) criteria for statistical significance (R^2^ value increasing by 1% or more), and* ***(iv) use of cross-sectional data that limits the ability to infer causality.***” (lines 555-556).

<Minor points>

Methods

1. Line 213: Although the full term "Timed Up and Go" is mentioned earlier in the text, the corresponding abbreviation "tug" is not introduced in parentheses at that point. The abbreviation later appears without prior clarification, which may confuse readers. To improve clarity and maintain consistency, please introduce the abbreviation in parentheses when the term is first mentioned (i.e., "Timed Up and Go (TUG)").

Author Response: Thank for noticing this discrepancy. We have made the following changes.

Author Action: Instead of adding another acronym, we have modified the term “TUG” to the full description (timed-up-and-go) (line 226).
